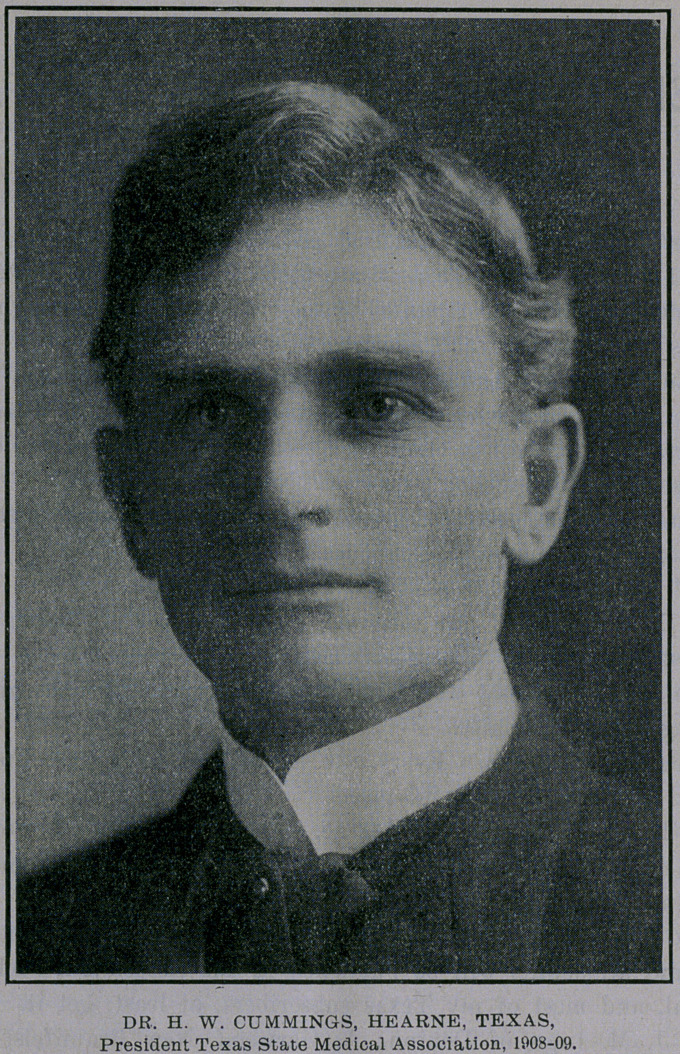# Echoes of the Corpus Christi Meeting

**Published:** 1908-06

**Authors:** 


### EDITORIAL DEPARTMENT.

### Echoes of the Corpus Christi Meeting.

There were over five hundred in attendance at the fortieth annual meeting of the Texas State Medical Association, and some fifty members were accompanied by their wives, sisters or daughters. It was the most enjoyable meeting ever held.

Beautiful Mrs. Wagstaff, of Corpus Christi, sang divinely.

The fish-fry on the beach was an epoch-marking event. Spanish mackeral, trout, redfish, flounders, cooked to tempt the epicure, were served with smiling grace by beautiful women.

Senator Willacy delivered a stirring address of welcome with his accustomed elegance and eloquence.

The presidential address of Dr. Cantrell was fine.

Dr. M. Duggan, of San Antonio, read an unusually strong and interesting paper on "The Social Evil."

The social functions were high class and much enjoyed—by the visiting ladies especially.

Harmony prevailed. Everybody seemed to be glad. Corpus Christi is a most delightful place.

Dr. H. W. Cummings, of Hearne, was elected President, defeating Knox, of Houston, by one vote. Cummings rose from the ranks and represents the "boys from the forks of the creek."

I do not enlarge upon the C. C. meeting because full proceedings and all details will be published in the Association's official journal, and most of my Texas subscribers, at least, get it.

Dr. J. M. Inge, of Denton; A. Garwood, New Braunfels, and Wood, of Hubbard, are the Vice-Presidents.

---

An Association of Health Officers was organized the day after adjournment of S. M. A. It is not an adjunct of the State Association,—has no connection with it. Dr. Brumby, State Health Officer, is President. There are sixteen Vice-Presidents, one for each congressional district. A Constitution and By-Laws was drawn up by Drs. E. B. Parsons, of Palestine; J. M. Andrews, of Wharton, and Young of Austin, and adopted. About one hundred members were enrolled. A full report appears in this issue. The several papers read will appear exclusively in the "Red Back."

DR. H. W. CUMMINGS, HEARNE, TEXAS,

President Texas State Medical Association, 1908-09.

Hatch Whitfield Cummings was born in Aberdeen, Miss., September 14, 1869, and was educated in the Mississippi public schools, receiving his M. D. degree from the Medical Department of the University of Tennessee, class 1892. He came to Texas in 1892 and began the active practice of medicine, which he has continuously followed to date. He organized the Brazos Valley Medical Society in 1894, and was its first President. He is a member of the Robertson County Medical Society, State Medical Association of Texas, and American Medical Association; Councilor (Texas State Medical Association) of Eleventh and later of Twelfth Districts. He was president of Hearne board of school trustees for seven years, and is vice-president of the Planters and Merchants State Bank at Hearne, and surgeon for the H. & T. C. R. R. and also I. & G. N. R. R., etc.

The "Red Back" for May created a sensation on account of two remarkable articles in it, "Social Hysteria," by Lydston, and a muchly-married woman's letter by "Mrs. Pluto." The letter will be answered in the "Red Back."

---

No Race Suicide in Texas.—Dr. Wilkins, of Wellington, in the Panhandle, reported that he and the two other doctors in his town caught three pairs of twins one night, and it was not a good night for twins, either. Dr. Holman Taylor, of Marshall, saw his ante and called him. He reported that he and his father caught six (single shots) in one night.

---

Galveston was selected as the next place of meeting, May, 1909, defeating Dallas, 16; Amarillo, 16; Galveston, 44.

---

Apropos of Galveston, in response to numerous requests the following, reproduced from memory, is published in the "Red Back":

When the House of Delegates came to select the place of meeting for the next annual convention, Dallas and Galveston, Waco and Amarillo were put in nomination. Dr. Karnes, of Dallas county made a ringing speech for Dallas, in which, after picturing in eloquent language the many inducements held out by that beautiful city, said that Dallas was the head of navigation and in telephonic communication with the moon,—a sentiment that was much applauded. Dr. Daniel, of Austin, arose and said:

"I file a brief for Galveston, and am here to present the claims and advocate the cause of the city by the sea.

"This Association has ever shown a regard for established precedent and the square deal. We have for many years swung around in a circle of seven points, meeting in each of the larger cities once in seven years. Eliminating Waco and Amarillo as unavailable, as I understand, Galveston comes in regular order and is *entitled* to the meeting. We have met in Dallas since we met in Galveston, and fair play demands that we now accept her courteous and cordial invitation. We met there in response to her invitation at a time when she was not yet even fairly convalescent from the cruelest blow ever struck by the forces of nature to a slumbering and unsuspecting people. We met there when she was paralyzed, because, even then, she desired to testify to the world and to the medical profession especially, her appreciation of the generous and tender sympathy that poured in upon her from all parts of this great State, and she did her best to entertain us and make us glad. And she succeeded,—to our satisfaction, but not to hers. Crushed and bleeding, helpless and forlorn, like outraged Innocence, she lay. But, thanks to the courage and virility of her splendid citizenship, she rose, like the fabled Phœnix, from the ashes of her desolation. Like Anteus of old, rejuvenated and reinvigorated, she shook herself and is now invincible. Her adamantine Front, like old King Canute, now defies Neptune, and like him says, 'Thus far shalt thou go, and no further.' She says, 'Come! Come to our beautiful new city, risen, like rosy Venus, from the sea foam! Come to our homes and to our hearts, and let us tell you how we love the noble, generous, self-sacrificing doctors of Texas! Come! Come from the rock-ribbed hills and from the smiling plains now blooming in their beauty and budding with promise of the products that feed and clothe the world! Come from the mesquite hammocks and the cross-timbers and the Staked Plains and the Panhandle of Texas. We would do you honor. We would send you home with a song in your heart like that of the conch shell which, torn from its home on

Some distant starlit isle,
Where rhythmic wavelets break o'er silv'ry sands, carries bright memories in its heart and ever sings the sad song of the sounding sea,—and we pray that you never forget us!'

"Can you resist that appeal, my colleagues? Can you deny her—in the language of the boys—'a show for her white alley?' God forbid! Let us gladly go. Let us make her glad. Let us enjoy the feast of reason and the flow of soul she is storing up for us! We have come to beautiful Corpus for the first time. We have breathed the sea breezes, salt-laden and salubrious, and have drunk in deep draughts of health. We have feasted on the dainty sea trout and the flounders and the mackerel and the snapper, and, like Oliver Twist, I for one, 'want more.' Let us go! There, every facility for the work to be done will be afforded us. There, is our great school of medicine, the pride of Texas. Its grand buildings with spacious halls, corridors and pavilions will be turned over to us, and our House of Delegates, our sections and joint sessions can be held under one roof and in adjoining rooms. Sandwiched between meetings, bathing in the surf, receptions adorned by the beauty, wit and grace of her matchless women; dances for the gay and banquets for the convivial, and speech-making and toasts and responses for all,—and back seats for none; sails on the bay, fishfrys on the beach, and, oh! the delicious oyster roasts down the Island. All these await us! The memory of them still lingers with us and the anticipation makes my mouth water.

"The eloquent gentleman from Dallas boasts that that city is the head of navigation and in telephonic communication with the moon! Let it not be forgotten that Galveston is the *source* of navigation and in telepathic touch with the planet Mars, and that she is giving the Martians pointers on seawall building and canal digging! And when the cut-off at Panama shall have been completed and the Pacific ocean brought to our very doors, Galveston will become the greatest seaport in the world, and her teeming millions will embrace representatives of every nation under the sun! Let us go to the seashore. The beautiful city beckons us. Her citizens invite us. Our brethren beseech us, and the lovely women lure us with smiling eyes!"

On the show-down Galveston won out, 44 to 16.

---

THE "Red Back" always has something out of the ordinary,—short and sharp, and strong papers by able writers, worth preserving. Those who do not get it—get left. Subscribe now. Volume XXIV begins next month, July.

---

NOTICE.—I desire to buy an unopposed practice, and will buy property if same suits me. West of San Antonio, south of the T. & P. R. R. Temperance place. Have cash to pay for what suits me. Will want possession November. Write all particulars, especially roads, churches, lodges, nationality of patrons, etc. Address J. L. G., TEXAS MEDICAL JOURNAL, Austin, Texas.

## Figures and Tables

**Figure f1:**